# Differential Effects of Linagliptin on the Function of Human Islets Isolated from Non-diabetic and Diabetic Donors

**DOI:** 10.1038/s41598-017-08271-9

**Published:** 2017-08-11

**Authors:** Yanqing Zhang, Meifen Wu, Wynn Htun, Emily W. Dong, Franck Mauvais-Jarvis, Vivian A. Fonseca, Hongju Wu

**Affiliations:** 1 Department of Medicine, Tulane University School of Medicine, New Orleans, Louisiana USA; 2 Department of Medicine, Guangdong Medical University, Zhanjiang Guangdong Province, China

## Abstract

Linagliptin is a dipeptidyl Peptidase-4 (DPP-4) inhibitor that inhibits the degradation of glucagon-like peptide 1 (GLP-1), and has been approved for the treatment of type 2 diabetes (T2D) in clinic. Previous studies have shown linagliptin improves β cell function using animal models and isolated islets from normal subjects. Since β cell dysfunction occurs during diabetes development, it was not clear how human islets of T2D patients would respond to linagliptin treatment. Therefore, in this study we employed human islets isolated from donors with and without T2D and evaluated how they responded to linagliptin treatment. Our data showed that linagliptin significantly improved glucose-stimulated insulin secretion for both non-diabetic and diabetic human islets, but its effectiveness on T2D islets was lower than on normal islets. The differential effects were attributed to reduced GLP-1 receptor expression in diabetic islets. In addition, linagliptin treatment increased the relative GLP-1 vs glucagon production in both non-diabetic and diabetic islets, suggesting a positive role of linagliptin in modulating α cell function to restore normoglycemia. Our study indicated that, from the standpoint of islet cell function, linagliptin would be more effective in treating early-stage diabetic patients before they develop severe β cell dysfunction.

## Introduction

The islets of Langerhans in pancreas play essential roles in regulating blood glucose homeostasis. Each islet is typically composed of 5 types of endocrine cells, which include the glucagon-producing α cells, insulin-producing β cells, somatostatin-producing δ cells, ghrelin-producing ε cells, and polypancreatic peptide-producing PP cells. Among them, β and α cells, acting through their respective hormones, are most critical in the regulation of blood glucose. Insulin stimulates the storage of glucose as glycogen and triacylglycerol in various tissues, thus reducing blood glucose levels, whereas glucagon activates glycogenolysis, ketogenesis and gluconeogenesis when blood glucose is low. A tight paracrine regulation between insulin and glucagon is present in normal conditions, in which an increase in insulin suppresses glucagon secretion, and a decrease increases it^1–3^. Development of diabetes is mainly attributed to the lack of insulin-producing β-cells (type 1 diabetes, T1D) or as a result of insulin resistance (type 2 diabetes, T2D) that eventually leads to β-cell dysfunction or failure. However, many observations suggest that glucagon perpetuates the development and progression of diabetes because in diabetic conditions, despite the presence of hyperglycemia, high level of glucagon (i. e., hyperglucagonemia) is observed^1, 4–6^, suggesting the regulation of glucagon secretion is impaired.

The glucagon-like peptide 1 (GLP-1) is a 36 amino acid peptide hormone encoded together with glucagon by the proglucagon gene. Originally discovered as a gut hormone secreted by the endocrine L-cells in the small intestine and colon, GLP-1 acts on multiple organ systems to regulate nutrient ingestion and blood glucose^7–14^. In the pancreas, GLP-1 stimulates insulin secretion from β-cells in a blood-glucose dependent manner, while inhibits glucagon secretion from α-cells, thus playing an essential role in blood glucose regulation^15, 16^.The physiological properties of GLP-1 have rendered it an attractive target for diabetes treatment. Because native GLP-1 is highly susceptible to degradation by Dipeptidyl-Peptidase IV (DPP-4), which is abundantly expressed in the endothelium of capillaries adjacent to the L-cells^16, 17^, two types of GLP-1-based drugs are developed. One is GLP-1 receptor (GLP-1R) agonists that mimic GLP-1 action but are resistant to DPP-4 degradation, and another is DPP-4 inhibitors that block GLP-1 degradation and thus prolong the activity of endogenously expressed native GLP-1.

Linagliptin (Tradjenta, Boehringer Ingelheim) is a specific DPP-4 inhibitor that is in clinic use for the treatment of T2D patients^18, 19^. Studies have shown linagliptin increases GLP-1 concentrations in blood and improves β cell survival and function^20, 21^. In addition, since GLP-1 is also produced locally within pancreatic islets, and its expression increases with the progress of diabetes development^22–27^, linagliptin may exert insulinotropic and glucagonostatic effects directly on the islets by elevating local GLP-1 concentrations. Indeed, Shah *et al*. have shown linagliptin significantly increases GLP-1 concentration in the culture media of human islets, and protects β cells from gluco-, lipo-, and cytokine-induced toxicity^28^. However, it is well documented that β cell dysfunction occurs with T2D development, in which β cells gradually lose their characteristic markers such as transcription factors and functional proteins, and become glucose insensitive^29, 30^. Therefore, islets of diabetic patients may respond to linagliptin treatment differently from normal human islets. This study is thus designed to evaluate the direct effects of linagliptin on human islets with different diabetic conditions. To accomplish this, we employed human islets isolated from non-diabetic and diabetic donors, and examined whether linagliptin affects their hormone secretion including GLP-1, glucagon, and insulin.

## Results

### Expression of GLP-1 and DPP-4 in human islets supported a local effect of linagliptin on islet cell function

**Figure 1:**
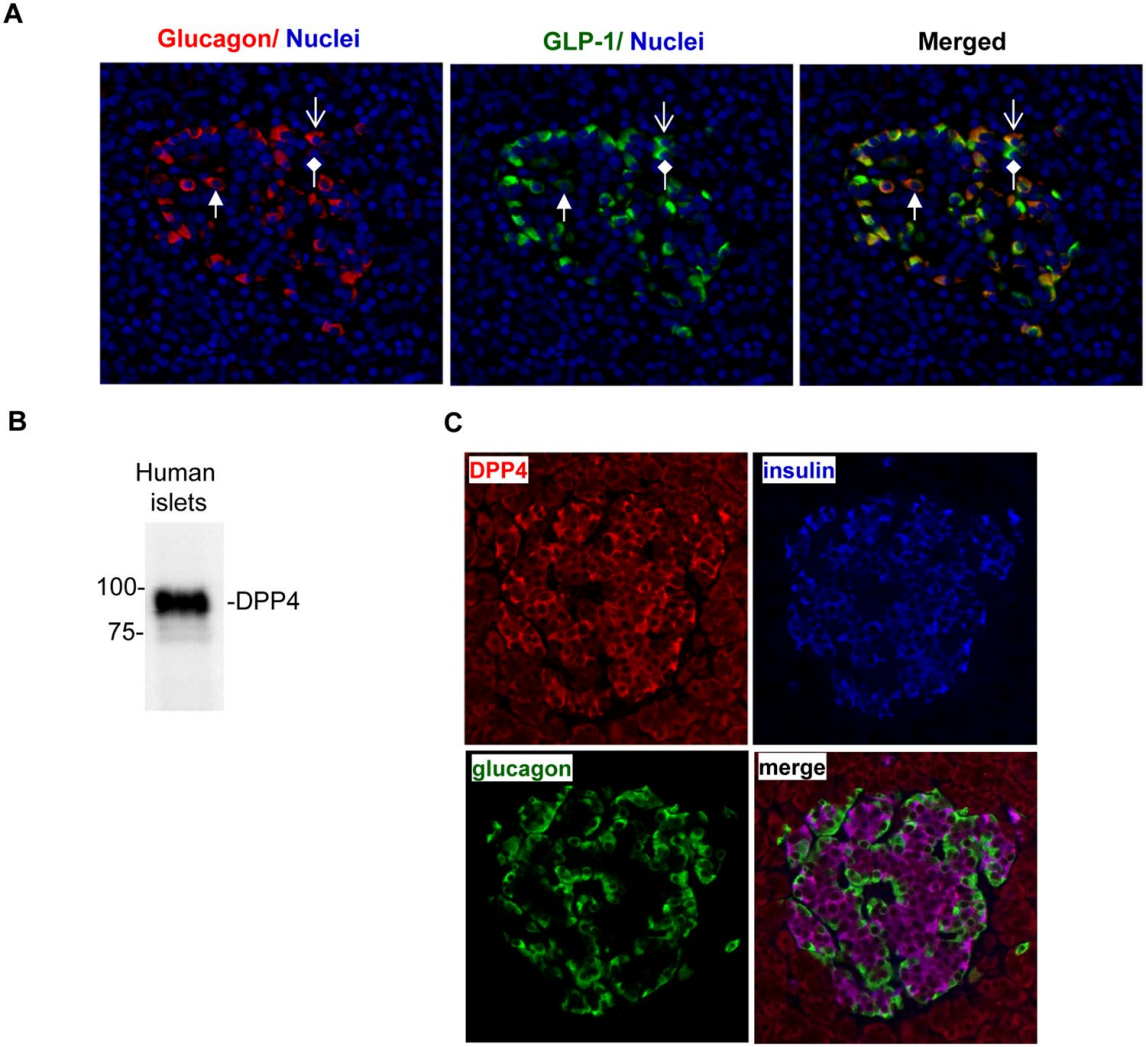
Expression of GLP-1 and DPP-4 in human islets. (**A**) Immunofluorescence staining showing GLP-1 expression in pancreatic islets. Normal human pancreatic slices were co-stained with anti-glucagon and anti-amidated GLP-1_7–36_ antibodies. DAPI staining (blue) was used to mark nuclei. Shown are representative images of >20 islets. The open arrow marks an example of cells expressing both GLP-1 and glucagon; the block arrow marks one predominantly expressing glucagon; and the diamond arrow marks one predominantly expressing GLP-1. (**B**) Western blotting assay showing expression of DPP-4 in human islets. The assay was repeated 5 times with different batches of normal human islets. (**C**) Immunofluorescence staining of DPP-4 in normal human pancreatic tissue. Shown are representative images of >20 islets.

Classically deemed as a gut hormone, GLP-1 has also been shown to be produced locally within pancreatic islets as GLP-1 and glucagon share the same precursor proglucagon that is expressed by α cells^24–27^. Using a monoclonal antibody that specifically recognizes the cleaved and amidated C-terminal end of active GLP-1, we confirmed GLP-1 expression in the islets of human pancreatic tissue, in which GLP-1 and glucagon were co-detected in about 70% of α-cells (Fig. 1A). To determine whether linagliptin could affect local GLP-1 concentration, we examined whether DPP-4 was expressed in the islet cells. As shown in Fig. 1B, DPP-4 was detected in the lysates of human islets, which appeared as a highly specific band at expected molecular weight of 88 KDa. To determine its cellular localization, we performed immunofluorescence triple staining for DPP-4, insulin, and glucagon using human pancreatic slices. We found that DPP-4 was predominantly expressed in β cells, with much lower level expression in α cells as well as in exocrine cells (Fig. 1C). Intra-islet expression of GLP-1 and DPP-4 supported that linagliptin could have significant effects on islet cell function through protecting local GLP-1 from degradation.

### Effects of linagliptin treatment on basal insulin and glucagon secretion of human islets varied between diabetic and non-diabetic donors

Previous studies have shown beneficial effects of linagliptin on β cell survival and function in islets isolated from normal human donors^28^. Islets of diabetic patients, however, might respond to linagliptin differently. To test this, we employed human islets isolated from non-diabetic (ND, n = 5) and diabetic (T2D, n = 5) donors (Table 1), and incubated them with 0 (untreated), 5 nM, 30 nM or 50 nM linagliptin for 3 days with daily media refreshing. The culture media was collected and used to evaluate their basal hormone secretion under normal glucose concentration of 5.5 mM.

**Table 1:** Human islets donor information.

Donor	Diabetes status^*^	Age	Gender	Diabetes duration	Diabetes treatment	BMI^**^	HbA1c (%)**	BG (mg/dL)**	Cause of death**	Islet purity^#^	Islet viability^#^
1	ND	47	F	n/a	n/a	21.6	n/k	n/k	stroke	95%	95%
2	ND	40	M	n/a	n/a	36.5	n/k	n/k	stroke	80%	93%
3	ND	47	F	n/a	n/a	24.5	4.8	n/k	stroke	90%	90%
4	ND	27	M	n/a	n/a	24.5	5.5	n/k	anoxia	90%	98%
5	ND	45	F	n/a	n/a	21.5	4.8	134	head trauma	60%	98%
6	T2D	58	M	20 years	Oral, no insulin	31.2	5.7	n/k	stroke	50%	99%
7	T2D	58	F	33 years	insulin	36.3	4.9	n/k	stroke	80%	95%
8	T2D	59	M	n/k	insulin	32.5	6.6	184	stroke	90%	95%
9	T2D	30	M	<5 years	insulin	35.9	5.4	176	stroke	90%	95%
10	T2D	61	M	<5 years	no insulin	28.4	5.2	163	stroke	95%	95%

ND: non-diabetic; T2D: type 2 diabetic; n/a: not applicable; n/k: not known.

*: Diabetes status is based on doctors’ diagnosis on medical history.

**: Data obtained during terminal hospital stay. Blood glucose (BG) data means average BG during the terminal hospital stay.

#: Information provided by islet providers (IIDP centers).

**Figure 2:**
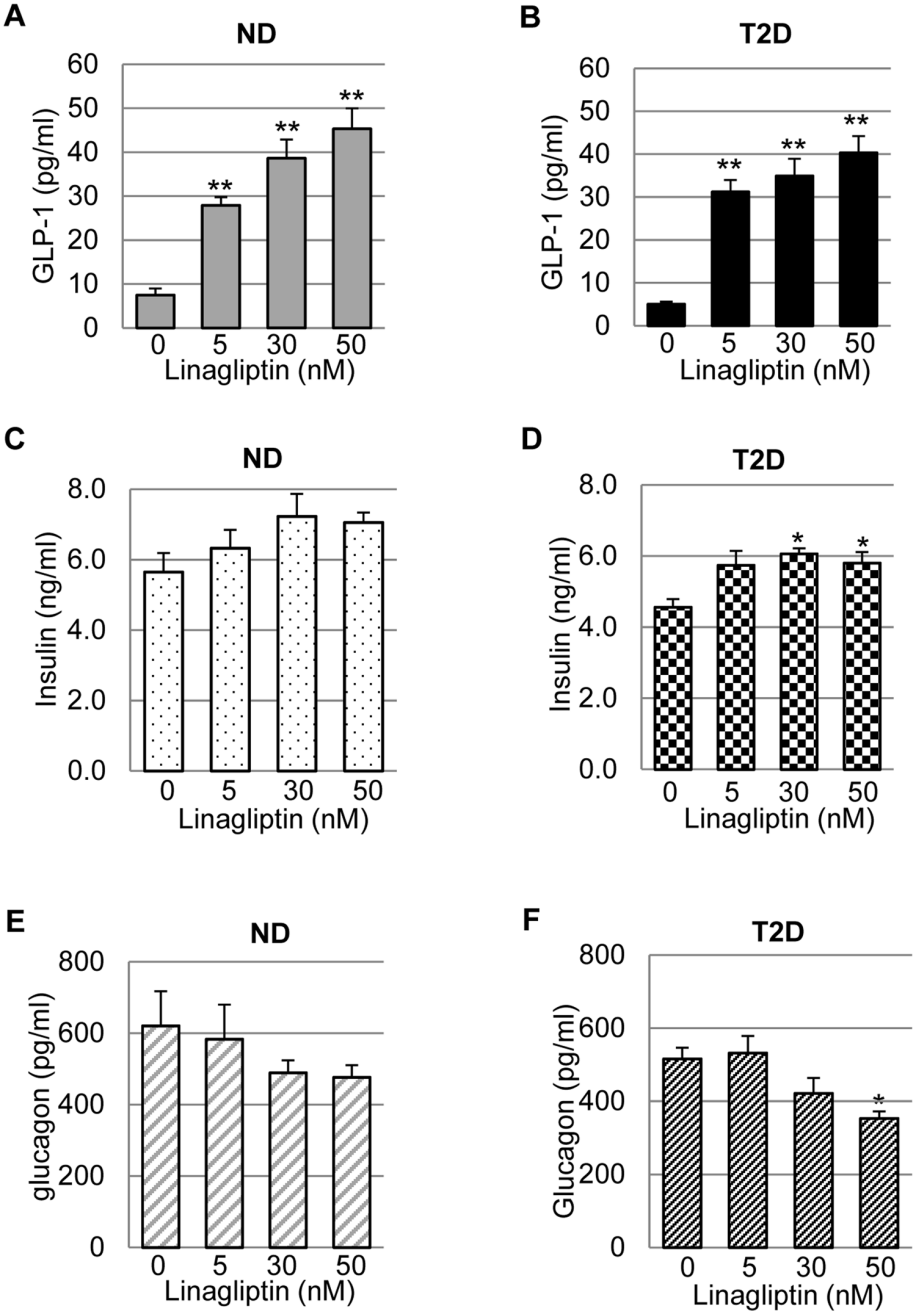
Basal GLP-1, insulin and glucagon secretion in cultured human islets following linagliptin treatment. Human islets isolated from ND and T2D donors were cultured at a density of 150 IEQ/0.5 ml in the presence of 0, 5, 30, and 50 nM linagliptin for 3 days with daily media refreshing. The media was collected and used to measure GLP-1 (**A**,**B**), insulin (**C**,**D**), and glucagon (**E**,**F**) concentrations using corresponding ELISA kits. Shown are representative data obtained from the 3^rd^ day culture. All data were normalized with the total protein of islet lysates that were collected at the end of the experiments (per ug protein), and expressed as Mean ± SEM. *p < 0.05; **p < 0.01 when compared to untreated group.

As shown in the representative sets of experiment (Fig. 2), linagliptin treatment significantly increased GLP-1 concentration in the culture media of both ND and T2D islets in a dose-dependent manner, with the highest concentrations reaching ~5 times of that in untreated groups (Fig. 2A,B). The dramatic elevation of GLP-1 in the islet culture had a tendency to stimulate basal insulin secretion (Fig. 2C,D) and to inhibit basal glucagon secretion (Fig. 2E,F), but the significance varied between normal and T2D islets. Specifically, normal islets did not significantly alter their basal insulin (Fig. 2C) or glucagon (Fig. 2E) secretion in response to linagliptin-mediated GLP-1 elevation. This was not surprising because the islets were cultured at normal glucose of 5.5 mM and studies have shown incretin effect on islet hormone secretion is glucose-dependent^31–34^. In T2D islets, however, basal insulin secretion was significantly stimulated and glucagon secretion significantly inhibited by linagliptin when its concentrations reached 30 nM − 50 nM (Fig. 2D,F). These data suggested that the glucose-dependent incretin responses diminished in T2D islets.

### Linagliptin improved β cell function with different efficacies in normal and diabetic islets

**Figure 3:**
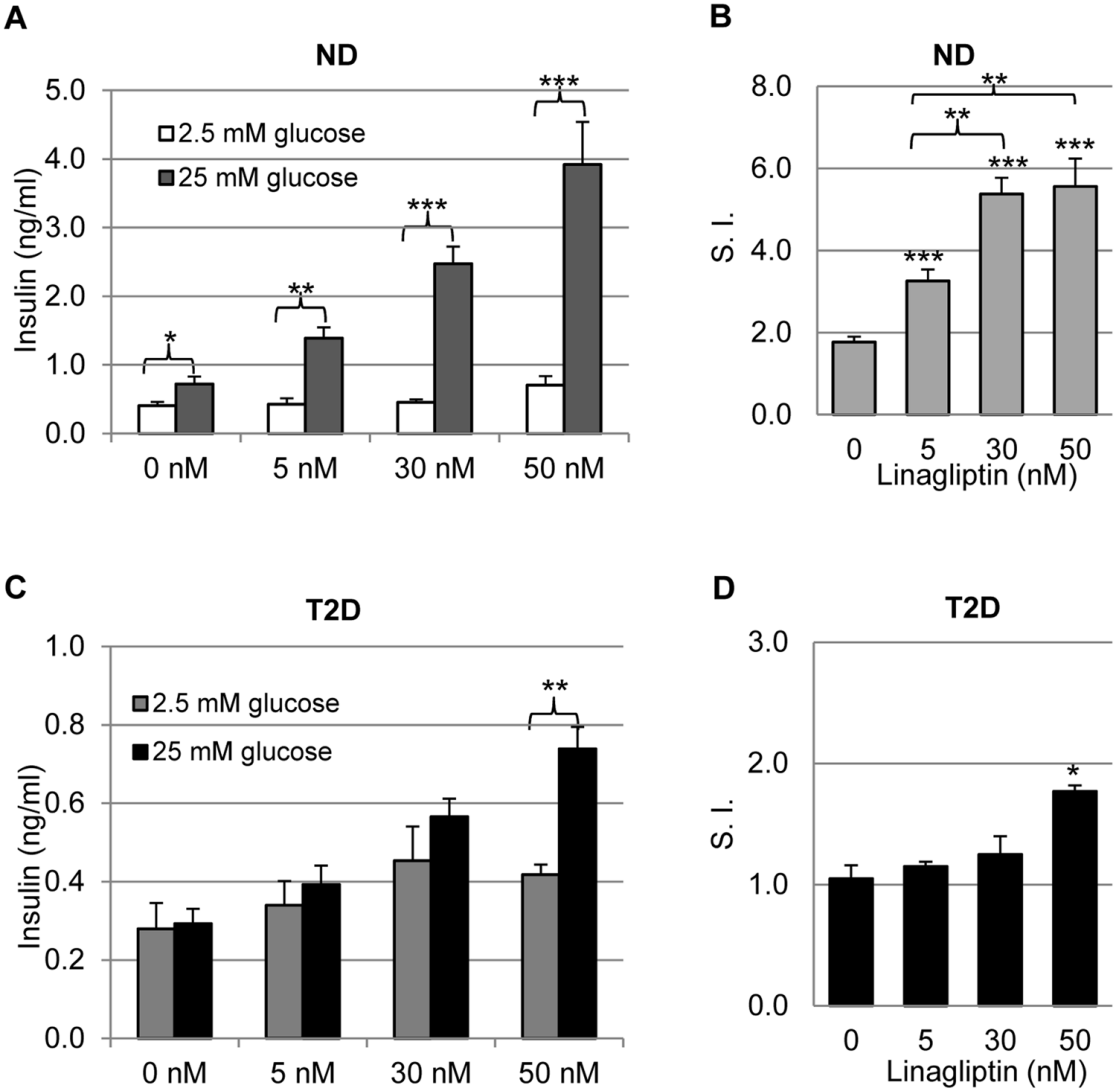
Glucose-stimulated insulin secretion in response to linagliptin treatment. ND (**A**) and T2D (**C**) human islets were subjected to GSIS tests using 2.5 mM as low glucose and 25 mM as high glucose conditions, in the presence of 0, 5, 30, and 50 nM linagliptin. All data were normalized with the total islet lysate proteins (per ug protein), and expressed as Mean ± SEM. The stimulation index (S. I.) was calculated by dividing the insulin concentration at high glucose by that at low glucose for each group (**B**,**D**). *p < 0.05; **p < 0.01; and ***p < 0.001 (compared to untreated group unless indicated).

Next we examined whether linagliptin affected glucose-stimulated insulin secretion (GSIS), a key feature of β cell function, in human islets of different diabetic conditions. The islets were incubated at either low glucose (2.5 mM) or high glucose (25 mM) in the presence of various concentrations of linagliptin, and insulin concentration in the supernatants was measured. Our data showed that, without linagliptin, non-diabetic human islets (n = 5) often had stimulation indices of 1.5–3.0, whereas T2D (n = 5) islets had stimulation indices of 0.7–1.5. As shown in the representative sets of experiment (Fig. 3), linagliptin was highly efficient in improving GSIS of ND islets, in which all linagliptin dosages significantly improved their stimulation indices (Fig. 3A and B). In contrast, the T2D islets only showed significant GSIS improvement at 50 nM (Fig. 3C and D), suggesting linagliptin was less effective in improving β cell function of T2D islets than that of normal islets.

**Figure 4:**
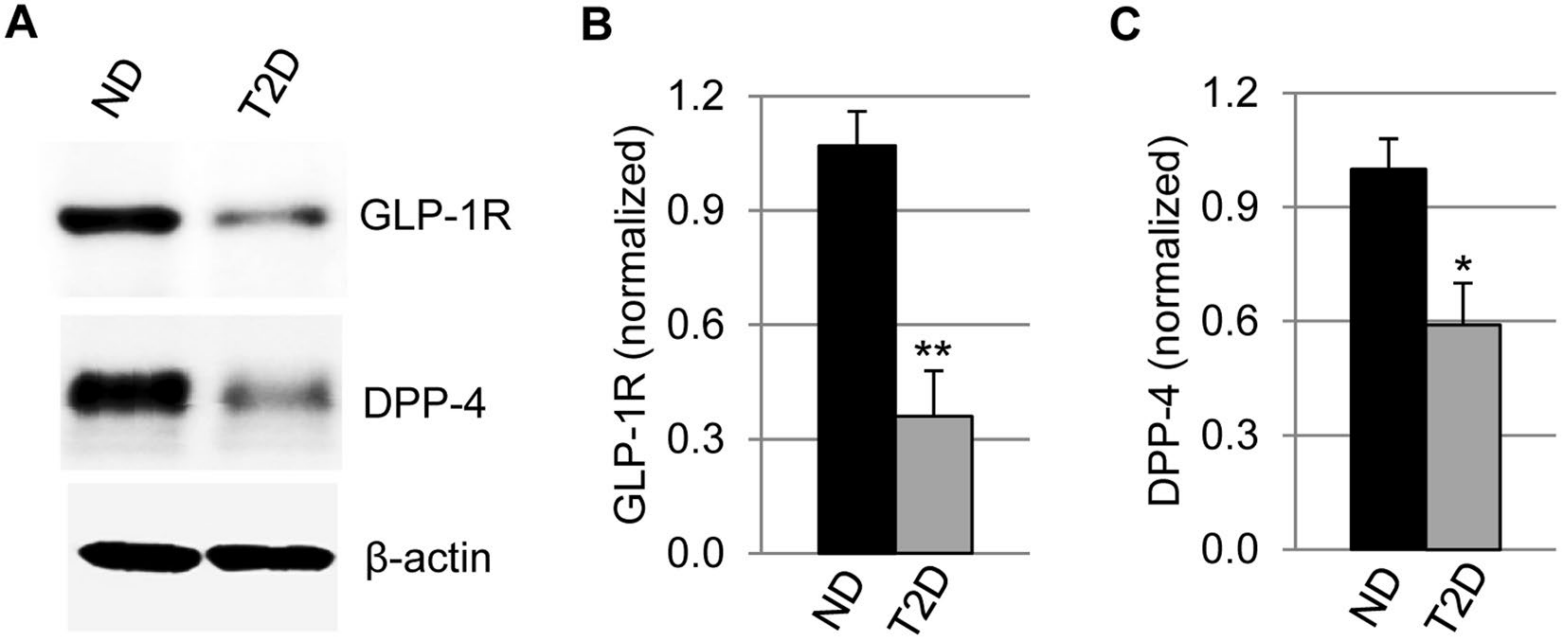
GLP-1R and DPP-4 expression in ND and T2D human islets. (**A**) Western blotting assay examining GLP-1R and DPP-4 expression in the lysates of ND and T2D islets. Beta-actin was included as loading control and reference for quantification. Shown are cropped images, and the full-length blots are included in the supplemental information. (**B**) Quantification of GLP-1R band signal that was normalized to β-actin signal (n = 5 for each group). (**C**) Quantification of DPP-4 signal normalized to β-actin (n = 5). The data were expressed as Mean ± SEM. *p < 0.05, and **p < 0.01.

Because linagliptin treatment resulted in similar GLP-1 elevation between ND and T2D islets (Fig. 2A,B), the reduced efficiency of linagliptin in T2D islets might be attributable to the change of GLP-1 receptor (GLP-1R) expression in the islets. Indeed, previous studies have shown GLP-1R expression in T2D human islets was significantly lower than that of normal islets^29, 30^. To confirm whether this was true in the human islets that were used in our study, we performed western blotting assays. Our data showed that GLP-1R expression was indeed significantly reduced in T2D islets compared to that in ND islets (Fig. 4A and B). DPP-4 expression also decreased in T2D islets (Fig. 4A and C), consistent with a previous study^35^.

### Linagliptin treatment led to increased GLP-1 versus glucagon production in human islets

**Figure 5:**
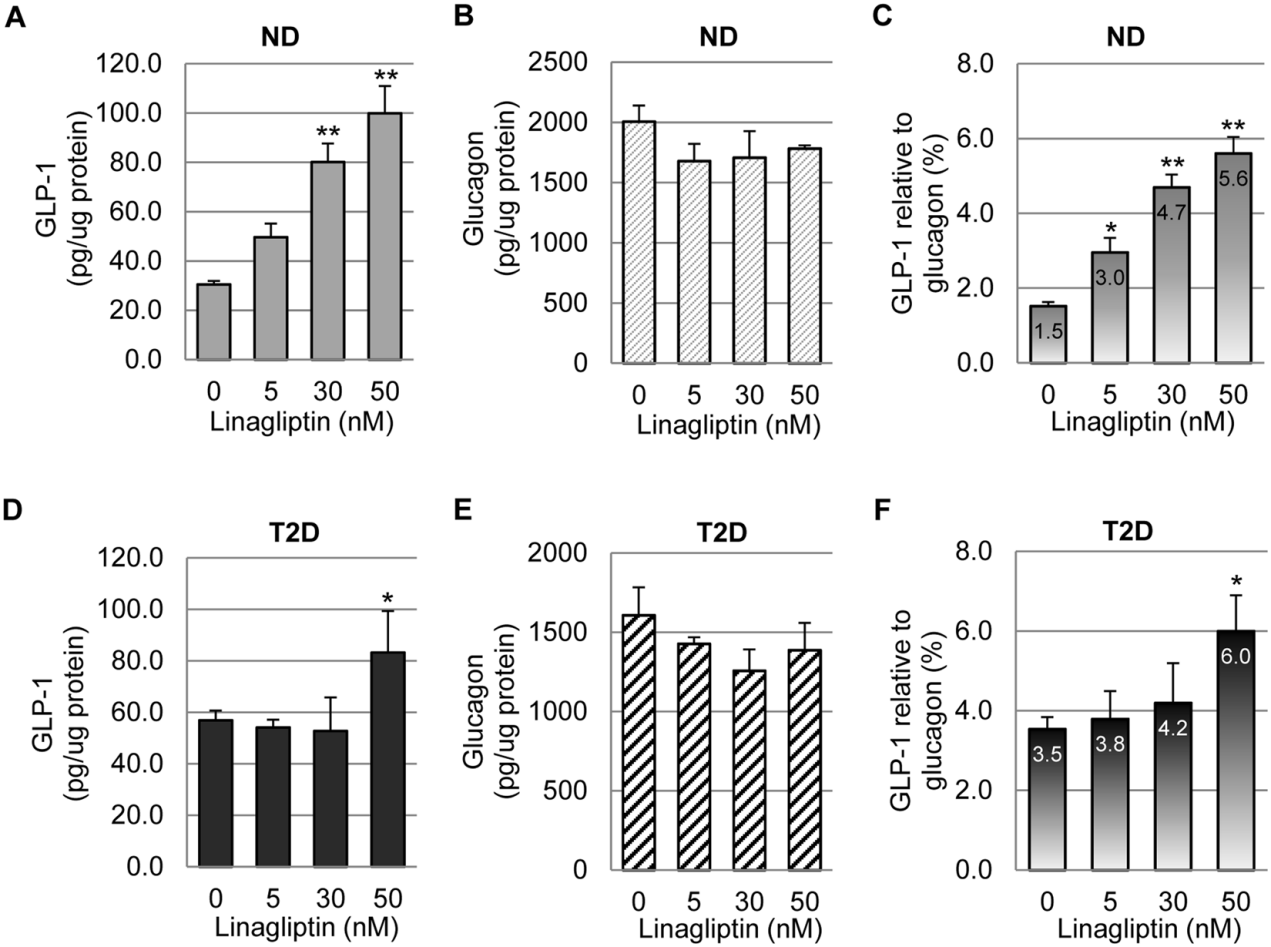
GLP-1 and glucagon production in human islets treated with linagliptin. Following 3-day linagliptin treatment, ND (**A–C)** and T2D (**D**–**F**) human islets were collected and lysed. GLP-1, glucagon, and total proteins were measured. The amounts of GLP-1 and glucagon were normalized with total protein. Relative GLP-1 to glucagon production was calculated by dividing the GLP-1 amount by the glucagon amount in the same sample, and then expressed in percentage format (**C**,**F**). *p < 0.05; **p < 0.01 compared to untreated group.

In addition to evaluating linagliptin effects on β cell function, we also investigated whether linagliptin affected α cell hormone production. It is known that proglucagon can be cleaved to produce either glucagon or GLP-1 via distinct post-translational processes in pancreatic islets, specifically by α cells^22^. Note: the term α cells herein was used to describe islet cells expressing proglucagon (instead of glucagon). We examined whether linagliptin had an effect on GLP-1 vs glucagon production in human islets of ND (n = 5) and T2D (n = 5) patients. To accomplish this, the human islets were treated with various concentrations of linagliptin for 3 days. Then the islet lysates were collected and processed for GLP-1, glucagon and total protein measurements. In ND human islets, without linagliptin treatment, GLP-1 production was ~30 pg/ug islet protein, whereas glucagon was produced at ~2000 pg/ug protein. This is consistent with previous studies showing proglucagon in pancreatic islets is processed to predominantly produce glucagon while a small portion is processed to produce GLP-1^36, 37^. Linagliptin treatment significantly increased GLP-1 production, for up to 100 pg/ug protein (Fig. 5A), and glucagon production trended lower without statistical significance (Fig. 5B). As expected, the relative GLP-1 to glucagon production was significantly increased by linagliptin treatment (Fig. 5C).

Similar pattern was observed in T2D human islets (Fig. 5D–F). However, the increase in GLP-1 production did not reach statistical significance until the highest linagliptin concentration tested (50 nM). We also noted that basal GLP-1 production in T2D islets (without linagliptin treatment) was ~60 pg/ug protein, nearly doubled compared to that of ND islets (Fig. 5D). This was in agreement with previous studies showing intra-islet GLP-1 production increases with T2D development^6, 21^.

**Figure 6:**
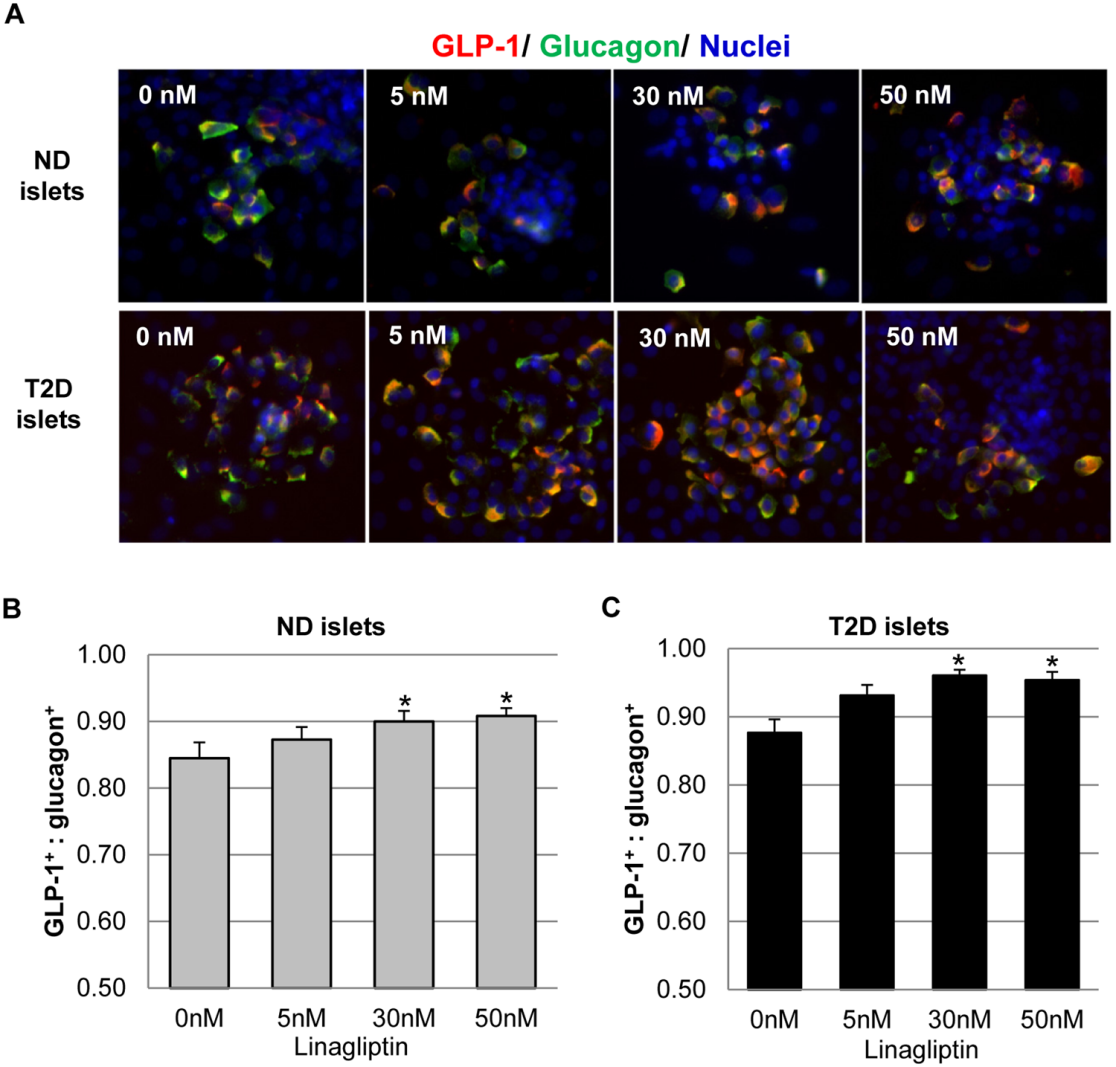
Immunofluorescence staining showing GLP-1 and glucagon expression following linagliptin treatment. (**A**) Representative images showing GLP-1 (red) and glucagon (green) expression in cultured human islets after 3 days of linagliptin treatment. (**B**,**C**) The ratio of GLP-1^+^ to glucagon^+^ cells in ND (n = 5) (**B**) and T2D (n = 5) (**C**) human islets. The numbers of GLP-1^+^ cells and glucagon^+^ cells were manually counted and the ratio was calculated for 25–30 islets in each group. The data were expressed as Mean ± SEM. *indicates *p* < 0.05 when compared to untreated control.

Furthermore, we examined GLP-1 and glucagon expression by immunohistochemistry following linagliptin treatment of ND and T2D human islets. GLP-1 and glucagon were co-expressed and readily detected in most α cells with or without linagliptin treatment (Fig. 6A). Quantification of GLP-1 positive cells (GLP-1^+^) and glucagon positive (glucagon^+^) cells showed increase in the ratio of GLP-1^+^: glucagon^+^ cells with increasing concentrations of linagliptin, in both ND and T2D islets (Fig. 6B). It should be noted that the number of GLP-1^+^ or glucagon^+^ cells did not reflect the amount of GLP-1 or glucagon produced in these cells due to the nature of the assays. For example, a cell would become GLP-1 positive in immunohistochemistry as long as its expression reached the detectable level. The increased number of GLP-1^+^ cells indicated that linagliptin treatment resulted in more α cells to express detectable GLP-1, which was in agreement with the increased GLP-1 production in these islets (Fig. 5)

## Discussion

DPP-4 inhibitors including linagliptin, sitagliptin, and vildagliptin have been shown to enhance β cell function of T2D patients as assessed by physiological parameters, and the effect is mainly attributed to upregulation of circulating GLP-1^38–40^. Little information was available regarding whether and how a DPP-4 inhibitor could directly affect islet cell function in diabetic patients. In this study, we examined this matter using human islets isolated from diabetic donors and compared it with those from non-diabetic donors. Our data showed that linagliptin significantly improved β cell function, but the effectiveness reduced in T2D islets compared to normal islets. The differential effects were attributable to reduced GLP-1R expression in diabetic islets, which agreed with β cell dysfunction during diabetes progression^29,30^. Furthermore, we examined whether linagliptin affected α cell function, and found that linagliptin significantly up-regulated the relative production of GLP-1 vs glucagon in α cells of both non-diabetic and diabetic human islets, which would be beneficial for restoring normoglycemia in diabetes treatment.

DPP-4 is a widely expressed protease. Omar *et al*. have recently shown DPP-4 is expressed in human islets, and its expression decreases with T2D development^35^, which is in agreement with our own discoveries. Nonetheless, there is some discrepancy: their study showed DPP-4 expression in α cells of human islets (and in β cells of mouse islets), while our data showed that DPP-4 was predominantly expressed in β cells of human islets. The DPP-4 antibody used in our study was commercially available and our western blotting assay confirmed that it was highly specific —it recognized one band at the expected molecular weight of DPP-4 in the full lane of whole islet extracts with little background staining (Fig. 1B and Fig. S2). The discrepancy with Omar *et al*. study is most likely due to the use of different antibodies and/or different staining protocols.

Linagliptin’s effects on normal human islets have been examined previously by Shah *et al*., in which the investigators have found that linagliptin restores β cell function through GLP-1 stabilization^28^. In addition, they have shown linagliptin has protective effects on human islets against cytokines-induced toxicity as well as gluco- and lipotoxicity that are often associated with diabetes development^28^. Our results on non-diabetic islets supported their discoveries. Furthermore, we obtained informative data regarding how the islets of diabetic patients responded to linagliptin directly. We found that linagliptin significantly increased GLP-1 concentration to the similar extent in both normal and T2D islet cultures despite different DPP-4 expression levels, but its effectiveness on improving GSIS differed. The phenomenon could be readily explained by the expression of GLP-1R in islets of patients at different stages of their diabetes. In some severe cases, β cells might be so dysfunctional that they wouldn’t respond to linagliptin at all. In fact, we did encounter such a case, in which even 200 nM linagliptin didn’t significantly improve β cell function in response to high glucose (supplemental Fig. S1). Together with β cell protection effect of linagliptin^28^, these results indicate linagliptin would be more beneficial and more effective when used in clinic for patients at early stage of T2D.

Another interesting discovery in the study is that linagliptin did not significantly affect basal insulin and glucagon secretion of ND islets, but did in T2D islets at normal culture condition, although GLP-1 levels were elevated to similar levels (Fig. 2). It is well documented that GLP-1 stimulates insulin secretion and suppresses glucagon secretion in a glucose-dependent manner^31–34^. Therefore, it is not surprising that basal insulin and glucagon secretion in ND islets was not altered by linagliptin-mediated GLP-1 elevation. In contrast, T2D islets might have developed certain degree of β-cell dysfunction so that the glucose-dependence of GLP-1 action was compromised in these islets. On the other hand, it should be noted that this is beneficial because increased insulin and decreased glucagon is good for lowering blood glucose in T2D patients.

Furthermore, although hyperglucagonemia is commonly observed in T2D patients, we did not detect any significant change in basal glucagon secretion in cultured T2D islets compared to normal human islets (Fig. 2). It should be noted that the mechanisms underlying glucagon dysregulation in diabetes are not completely understood, but appear to be attributable to many factors that are affected by insulin resistance and hyperglycemia. In particular, studies have shown that impaired paracrine insulin inhibition (due to insulin resistance)^1–3, 41^, impaired blood glucose-sensing in the α-cells^42^, and autonomic dysfunction in the nervous system^43, 44^, are involved in this process. In this study, the cultured T2D human islets did not show increase in basal glucagon secretion compared to normal human islets (Fig. 2). This could mainly because: (1) the islets were cultured at normal glucose concentrations, thus not affected by hyperglycemia; and (2) there was no effect from autonomic nervous system in isolated islets culture.

The effect of linagliptin on α cell function, specifically hormone production and secretion, was also investigated in this study. It has become increasingly clear that α cells produce not only glucagon, but also GLP-1, although the latter was produced at much lower level than the former^22–27^. In addition, studies have shown GLP-1 expression in α cells increases with T2D development, suggesting they play an integral role in the attempt to regain normoglycemia^22, 23, 26, 27^. Our results on untreated ND and T2D islets aligned well with these studies as we showed that GLP-1 was produced at ~1.5% of glucagon level in ND islets, whereas it was ~3.5% in T2D islets. Moreover, our data demonstrated that linagliptin significantly increased GLP-1 vs glucagon production in both ND and T2D islets. The number of α cells expressing detectable level of GLP-1 was also significantly upregulated by linagliptin in both ND and T2D islets. These data argue for a positive effect of linagliptin on α cells with regard to hyperglycemia alleviation.

In summary, our study provided important information regarding how linagliptin would affect islet cell function in diabetic patients when used in clinic. From the standpoint of islet cell function, linagliptin would be more effective in treating early-stage diabetes.

## Materials and Methods

### Antibodies

The rabbit anti-DPP-4 (CD26) polyclonal antibody (#ab28340), the guinea pig anti-insulin antibody (#ab7842), and the mouse anti-GLP-1 monoclonal antibody specific for the amidated C-terminus of the active form GLP-1_7–36_ (#ab26278) were purchased from Abcam (Cambridge, Massachusetts). The rabbit anti-glucagon (#2760 S) and the rabbit anti-β-actin (#13E5) antibodies were purchased from Cell Signaling Technology (Danvers, Massachusetts). The goat anti-GLP-1R polyclonal antibody (#TA326758) was purchased from OriGene Technologies (Rockville, Maryland). All of the secondary antibodies including anti-mouse, anti-rabbit, anti-goat, and anti-Guinea Pig antibodies, which were conjugated with either tetramethyl rhodamine (TRITC, red), fluorescein (FITC, green), coumarin (AMCA, blue), or horseradish peroxidase (HRP), were purchased from Jackson ImmunoResearch Laboratories Inc (West Grove, Pennsylvania).

### Human islets culture and treatment

Freshly isolated human islets were obtained from the Integrated Islet Distribution Program (IIDP) at City of Hope that was funded by National Institute of Diabetes and Digestive and Kidney Diseases (NIDDK, 1UC4DK098085). The donor and human islet information was listed in Table 1. The study was designated as “Not Human Subject Research” by the Institutional Review Board of Tulane University. Upon receiving the islet shipment, the islets were washed with fresh culture media (CMRL-1066 supplemented with 0.1 g/L L-glutamine, 10% FBS, 10 mM HEPES, 0.2% Sodium Bicarbonate, and 0.1 mg/ml ciprofloxacin), allocated into 48-well plates at a density of 150 Islet Equivalent Quantities (IEQ) per 0.5 ml per well, and cultured at 37 °C in a humidified incubator containing 5% CO_2_.

For linagliptin treatment, linagliptin (1 mg/ml stock solution) was diluted in the islet culture media and added into each well to achieve 5 nM, 30 nM or 50 nM final concentrations. Untreated islets (that is, 0 nM linagliptin) were included as control. The culture media was collected every day and replaced with fresh media containing the same concentrations of linagliptin, and cultured for up to 4 days.

### Hormone measurements

The concentrations of GLP-1, insulin and glucagon in the islet culture media were measured using corresponding ELISA kits from ALPCO Diagnostics (Salem, NH). Specifically, active GLP-1 was measured with GLP-1 (active 7-36) ELISA kit (cat# 43-GP1HU-E01), glucagon with glucagon (human, mouse, rat) ELISA kit (cat# 48-GLUHU-E01), and insulin with human insulin ELISA kit (cat# 80-INSHU-E01.1). All measurements were performed according to the manufacturer’s protocols. To determine the hormone expression (production) in the human islets after linagliptin treatment, the islets were washed with PBS, and lysed in 100 ul/well RIPA buffer containing a cocktail of protease inhibitors and phosphatase inhibitors. Following a freeze/thaw cycle, and 15 minutes’ incubation on ice, the samples were centrifuged at 4 °C, 13000 rpm for 10 minutes. The supernatants (cell lysates) were processed for hormone measurements as described above. In addition, total protein concentrations of the cell lysates were measured using Pierce BCA Protein Assay kit (Thermo Fisher Scientific, Waltham, MA).

### Glucose-stimulated insulin secretion

GSIS was performed essentially as described previously ^45^ except that various concentrations of linagliptin were included in Krebs-Ringer Bicarbonate (KRB) solutions that contained either 2.5 mM glucose (KRB-low) or 25 mM glucose (KRB-high). Specifically, following overnight culture after receiving the islets from IIDP centers, the human islets plated in 48-well plates (150 IEQ/well/0.5 ml) were pre-incubated in KRB-low solution for 30 minutes at 37 °C. Then the supernatants were replaced with either KRB-low with various concentrations of linagliptin (0, 5, 30, 50 nM), or KRB-high with various linagliptin concentrations (0, 5, 30, 50 nM), n = 4 wells per treatment group. Of note, 0 nM linagliptin depicts the islets without linagliptin treatment. After the plate was incubated for 1 hour at 37 °C in humidified 5% CO_2_ incubator, the supernatants were collected, and insulin concentrations measured as described above.

### Immunofluorescence staining

For isolated human islets, the islets were cultured and treated with linagliptin in collagen-coated 24-well plates for 4 days as described above. The islets were then fixed with 4% paraformaldehyde in PBS for 30 minutes, permeabilized with 0.25% Triton-X for 7 minutes, and blocked in blocking solution (2% Glycine, 2% BSA, 5% FBS, 50 mM NH_4_Cl in PBS) for 1 hour. The cells were then incubated with primary antibodies and fluorescence-conjugated secondary antibodies following standard protocols. For the paraffin-embedded human pancreatic slices, which were obtained from the network for Pancreatic Organ Donors (nPOD), de-paraffinization was performed prior to antibody staining using standard protocols^27^.

### Quantification of GLP-1^+^ and Glucagon^+^ cells

The numbers of GLP-1-expressing cells (GLP-1^+^) and glucagon-expressing cells (Glucagon^+^) in each islet was performed essentially as described before ^27^. Briefly, the GLP-1^+^ and Glucagon^+^ cells were manually counted from 25–30 islets in each group. The ratio of GLP-1^+^ cells (including GLP-1^+^ only and GLP-1^+^Glucagon^+^ double positive) to Glucagon^+^ cells (including Glucagon^+^ only and GLP-1^+^Glucagon^+^ double positive) was calculated for each islet, and averaged for each treatment group.

### Statistical analysis

The statistical analysis was performed using SAS software. One-way ANOVA was used to compare the differences among different treatment groups. In the cases involving only 2 groups, student’s t-test was used to determine whether the difference was statistically significant. The data were expressed as Mean ± SEM (standard error of the mean). *P* < 0.05 was considered statistically significant.

### Western blotting assay

Human islets (5000 IEQ) were lysed in 300 ul of RIPA buffer containing a cocktail of protease inhibitors and phosphatase inhibitors. After ultrasonic homogenization and a freeze/thaw cycle, the samples were centrifuged at 4 °C, 13000 rpm, for 10 minutes. The supernatants (protein extracts) were then transferred to fresh microcentrifuge tubes, boiled, and processed for SDS-PAGE and western blotting assays following standard procedures^46^.

### Data availability statement

The datasets generated during and/or analyzed during the current study are available from the corresponding author on reasonable request.

## Electronic supplementary material

Figure S1 and S2
