# Supplementary material for: Differential Effects of Linagliptin on the Function of Human Islets Isolated from Non-diabetic and Diabetic Donors
Source: Sci Rep. 2017 Aug 11;7:7964. doi: 10.1038/s41598-017-08271-9 (PMC5554162; doi:10.1038/s41598-017-08271-9)
Supplement: Supplementary file 1 — Figure S1 and S2 [file 41598_2017_8271_MOESM1_ESM.pdf]

Differential Effects of Linagliptin on the Function of Human Islets Isolated from Non-diabetic and Diabetic Donors

Yanqing Zhang, Meifen Wu, Wynn Htun, Emily W. Dong, Franck Mauvais-Jarvis, Vivian A. Fonseca, and Hongju Wu

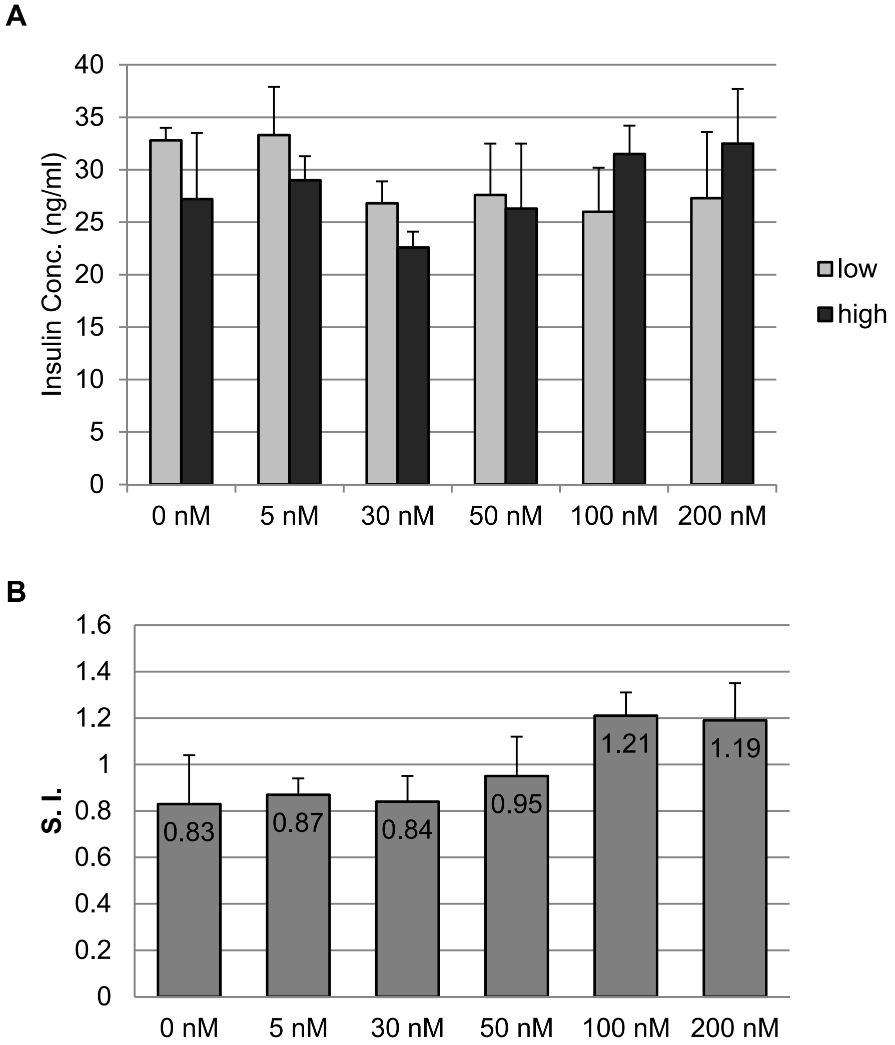

Figure S1: An example of T2D human islets showing very low responsiveness to Linagliptin. Glucose stimulation of insulin secretion (GSIS) was performed for the islets in the presence of various concentrations of Linagliptin that were cultured in 48-well plates at 150 IEQ/0.5ml/well, 4 wells/group. Stimulation Index (S.I.) was calculated by dividing the average insulin concentration at high glucose (25 mM) by that of low glucose (2.5 mM). Linagliptin (up to 200 nM) did not show significant effect on β cell function, suggesting these islets may have developed severe β cell dysfunction.

Differential Effects of Linagliptin on the Function of Human Islets Isolated from Non-diabetic and Diabetic Donors

Yanqing Zhang, Meifen Wu, Wynn Htun, Emily W. Dong, Franck Mauvais-Jarvis, Vivian A. Fonseca, and Hongju Wu

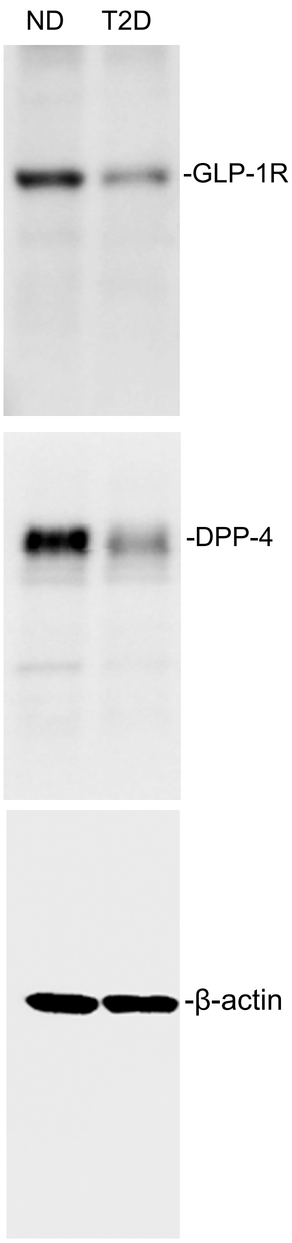

Figure S2: Original full-length gels for western blotting data in Figure 4.
